# Employing AI to Better Understand Our Morals

**DOI:** 10.3390/e24010010

**Published:** 2021-12-21

**Authors:** Luís Moniz Pereira, The Anh Han, António Barata Lopes

**Affiliations:** 1 Faculdade de Ciências e Tecnologia, Universidade Nova de Lisboa and NOVA-LINCS FCT/UNL, 2829-516 Caparica, Portugal; 2 School of Computing, Engineering and Digital Technologies, Teesside University, Middlesbrough, Tees Valley TS1 3BX, UK; T.Han@tees.ac.uk; 3 ANQEP—Agência Nacional para a Qualificação e Ensino Profissional, Agrupamento de Escolas de Alvalade, Av. 24 Julho, n.º 138, 1399-026 Lisbon, Portugal; lopesab@msn.com

**Keywords:** artificial intelligence, machine ethics, human morality, evolutionary game theory, AI governance

## Abstract

We present a summary of research that we have conducted employing AI to better understand human morality. This summary adumbrates theoretical fundamentals and considers how to regulate development of powerful new AI technologies. The latter research aim is benevolent AI, with fair distribution of benefits associated with the development of these and related technologies, avoiding disparities of power and wealth due to unregulated competition. Our approach avoids statistical models employed in other approaches to solve moral dilemmas, because these are “blind” to natural constraints on moral agents, and risk perpetuating mistakes. Instead, our approach employs, for instance, psychologically realistic counterfactual reasoning in group dynamics. The present paper reviews studies involving factors fundamental to human moral motivation, including egoism vs. altruism, commitment vs. defaulting, guilt vs. non-guilt, apology plus forgiveness, counterfactual collaboration, among other factors fundamental in the motivation of moral action. These being basic elements in most moral systems, our studies deliver generalizable conclusions that inform efforts to achieve greater sustainability and global benefit, regardless of cultural specificities in constituents.

## 1. Introduction and Background

Methods from statistical physics find applications in studies of human cognition and behaviour at different levels of organizations, from individuals to large groups, e.g., [1,2]. The present paper applies such tools in modelling morality with the help of AI. Modelling existing human solutions statistically about those most often adopted in the resolution of different moral dilemmas might lead to the perpetuation of evolutionarily sub-optimal decisions. Although statistical treatment of available historical data can highlight accepted solutions to a given dilemma—as preferred in each society, for a generation or according to race and ethnicity, for example—consensus is not a reliable criterion for discerning the right thing to do. At the same time, application of criteria derived from established moral theories, for instance those derived from Kantian or egalitarian principles, can be expected to exacerbate nascent injustices. The presently reviewed series of our studies looks past such limitations, leveraging the power of Evolutionary Game Theory (EGT) [3] to computationally model emergent stable states resulting from large-scale cooperative dynamics, as moderated and constrained by cognitive capacities including those of apology, recognition of intention, promise-keeping, and others.

Experimental studies that are the subjects of the present review have been carried out with the explicit goals of both understanding morality in a way that realistically represents moral agency in human beings, while avoiding problems resulting from the employment of such an understanding, especially in terms of informing social policy. To these ends, our studies, using ever more realistic constraints, are ongoing [4,5,6]. Other research has shown that distinct kinds of unselfish behaviour, encompassing cooperation, altruism, truth-telling, altruistic punishment, and trustworthiness, are explained better by preferences for following one’s personal moral standards on what is right or wrong to do in a situation [7].

One approach to modelling group-level morality applies certain views of ethical life, or one or another feature of a given moral theory and may be considered broadly a top-down based one. In such a case, a model is constructed that is governed by principles taken to be fundamental prior to a model-enhanced exploration of possibilities. For instance, a theory of rationality may constrain an “ethical” module, which subsequently directs the function of the model system to aim for given objectives (such as prudence or safety). Bottom-up approaches go in the opposite direction, and appear more promising and more intuitive, as they represent ethics as emergent rather than with principles predetermined in a top-down fashion. In the context of the individual human mind, Turing coincidentally suggested that research should focus on mental development, and not on the completed adult mind. The presently reviewed suite of studies likewise focuses on social learning of evolving individual agents, as constituents of large groups seeking equilibrium in the context of different environments, over intergenerational and evolutionary timespans, because one expects that ethics is a consequence of moral cognitive capacities that emerged in the development of our species [8,9].

Each of our approaches sheds light on morality, including the ways in which it may be idealized. However, the most promising models should be those respecting the influences of individual moral self-development and represent the dynamics through which individual innovations inform group-level stability by sustainable social strategies. With such dynamics in mind, we can begin to formulate an explicit conception of moral autonomy as a capacity made possible by the employment of counterfactual reasoning (knowing what I know today, what would I have done differently?), when exercised in a social-historical learning context over evolutionary time. In this way, computational models of morality can both expose the limits of our understanding of ourselves and allow us to move past its limitations, to avoid the pitfalls of past and current mistakes, including those which statistical treatments might only reinforce. In this direction, we move next to review various dimensions central to the research that has been our focus.

Prior work of ours uses techniques of logic programming to model individual capacities for morality sans emotion, including, for instance, abduction, integrity constraints, preferences, argumentation, counterfactual reasoning, dual-process reasoning, and belief revision and updates (for a review see [10,11,12]. At the social-cultural level, again sans emotions, we apply EGT. With EGT, our models have demonstrated that different cognitive capacities, including the ability to recognize intentions, to form and to dissolve commitments, to exact revenge, to issue apologies, to forgive, to express guilt, and to act through counterfactual reasoning, whether by themselves or in combination, reinforce the stable group dynamics that depend on achieving cooperation in populations of constituents with diverse strategies, with otherwise potentially exclusive interests. In this work, dynamics are also compared with those of populations in which such individual competencies are absent and resultant group-level stabilities fail to emerge [13]. Some of these explorations of emergent morality are summarized below, synoptically. Full details can be found in their respective focused publications, as indicated by the references cited in each specific context.

The EGT coordination games that we use, among others, have a singular connection to complex systems and to the dynamics of populations in social settings and to other problems of a nature distinct from our ethical one. In this respect, the works of Schelling and Skyrms are paramount in contemplating dynamical models of coordination: cf. references [14,15,16] of [17]. Some other applications involve so-called signaling games, utilized to bring to light the dynamics of transfer of information, and evolution of languages and their meaning: cf. references [14,15,18,19] of [17]; plus, games addressing problems of information communication plus coordination: cf. references [19,20,21,22,23] of [17]. This spate of problems concern information production and its exchange, and the concomitant entropy variation: cf. reference [24] of [17]. For instance, in signaling games, information content in signals identifying states has been measured in terms of the Kullback–Leibler distance: cf. reference [19] of [17]. However, herein we are most interested in those changes that take place all along the self-organizing trajectories of populations of individuals expressing a limited number of cognitive and strategic behaviours. 

## 2. Evolutionary Game Theory (EGT)

EGT provides a scaffold for our studies into the emergence of human morality. Game Theory was originally focused on Economics and was later applied in the context of the Cold War [15,25]. Subsequently, EGT arose to model the evolutionary dynamics of populations with social learning and mutations, with by now well-established basic techniques [3]. Its applications show that when social situations become complex, so that they cannot otherwise be resolved by unaided human reasoning, relatively straightforward analytical mathematics—plus, like in our work’s developments, the use of computer simulations whenever uncertainty caused by noise is involved—the multifaceted EGT is indispensable for clarifying the population dynamics. In the context of the emergence of human-like moral facets in populations, we consider that genes (and, by extension, memes as “cultural genes”) represent evolving strategies in evolutionary games. With this approach, we interpret moral and ethical problems in terms of survival of strategies that outcompete with others for a stable invasion of the whole population for a significant time. With computational models built on EGT principles, we observe the combinatorial evolution of individual strategies and their mutations under various conditions. They include differences in co-player partners, resulting in diverse offspring frequencies, given the rules of distinct culturally specific social games, or changes in natural contexts and constraints, for example.

Games involve uncertainty. To minimize the impact of uncertainty on the possibilities for survival, cognitive agents establish strategies to order the otherwise apparent disorder with action routines that result in reliable success, vis-à-vis their partner players. The development of such routines and analysis of the relative game player payoffs, can be studied in a formal mathematical way using EGT implemented in and simulated with computer programs. In general, a distinction can be made between ‘zero-sum’ and ‘non-zero-sum’ games. ‘Zero-sum’ are those in which some players lose exactly what others win. Robert Wright [26] analyses cultural evolution, determining that ‘non-zero-sum’ games are possible, which may conduce to win–win civilisation developments. In evolution in the natural world, according to Wright, conditions are not ‘zero-sum’—it is possible that everyone wins, or everyone loses—and that (most) everyone can win by cooperating through what he calls an enlightened reciprocal altruism. Reciprocal altruism involves one organism (temporarily) diminishing its own fitness to (temporarily) increase another’s at its expense and underwrites cooperation between non-related (non-kin) group members in “moral animals” like human beings. In other instances, diverse strategies active in an evolutionary context may establish a stable dynamic equilibrium that results in the balanced perpetuation of them all, even where they are at odds with one another, without altruism being enacted. Such is the classic relationship between predator and prey. The predator cannot over-consume the prey, and the prey cannot over-multiply at the expense of the limited supporting natural ecosystem. Either strategy, if not balanced by the other, would result in the extinction of both. Such lessons from the general study of games inform inquiries in diverse disciplines. For example, in Economics, those lessons help economists understand interactions under certain policy arrangements, such as in the infamous “tragedy of the commons” [23].

In any given game, interactions may occur just once with the same partner, or be iterated multiple times with the same or different partners. In this latter context, other questions present themselves, including the role of memory about specific partners, and how such experiences influence interactions with others because of their ascribed reputation [27], and whether refusing a partner based on past lessons is possible or even optimal in each context. With such considerations in mind, let’s begin by looking at the Prisoners’ Dilemma (PD), a game with a structure that typifies the “altruism paradox.”

In the classic PD, prisoners A and B are jointly held in suspicion of having committed a crime. A does not know what B may or may not be confessing, and vice versa. Either can confess to the crime or else say nothing (cf. the payoff Table 1 below).

As evident from the 2 × 2 payoff table, if both confess then both get eight years in prison. If one confesses and the other does not, then the confessor gets two years and the other ten. If both say nothing, then both get six years. It can be advantageous to not remain silent. Indeed, if one keeps silent while the other confesses, the confessor will be imprisoned for only two years while the silent one for ten: only the confessor gains. At worst, at the collective level, both confess and receive eight-year sentences, neither wanting to assume the risk of remaining silent. Yet, if both will remain silent, then each would receive only six years, as opposed to the eight years for each when both confess.

The PD is a classic game in which both players tend to confess. Unable to communicate, they cannot cooperate in achieving the more favourable result of the two remaining silent. Due to this uncertainty, players of the PD tend to suffer more than they might if there were some further avenues for cooperation available to them. Such avenues emerge as “honour amongst thieves,” for the PD can be played over many iterations with players remembering how partners played in the past. Betrayals may be met with revenge, or with intolerance and unwillingness to engage with that betrayer during future iterations.

Building on the classic PD, consider a multiplayer situation in which we ask what the best of all possible strategies over many iterations will be, adding just the sort of complexity that can be clarified with the help of a computer analytical computation or simulation. One starting assumption of such a model is that any strategy can play against any other, and from the memory of different experiences can then move on to other situations in which a given player will want to interact only with certain other players which proffer strategies that had not resulted in betrayals to them in the past. Thus, the simple PD can represent social cognitive constraints as parameters, thereby becoming more realistic.

Further adding to the realism, strategies may evolve by copying those of other augmented winning strategies (such as might be the case with statistical treatments of similar dynamics). Top winners may be understood as those that are able to reproduce more of themselves on their own. That is, they engender new players that are copies of themselves directly, rather than change their mind and strategy through experience gained by the iterations of structured engagement with diverse others. That is, the more successful have more “children.” Moreover, we may constrain the game by limiting the total fixed number of individuals that can be supported in a population, because, say, global environmental resources are limited. Therefore, some individuals are disposed of randomly with some probability, or according to some other condition or constraint. One option is that those who lose more (or gain less) are eliminated. Without sufficient resources, some will find themselves unable to reproduce, with their represented strategies subsequently dying out. Under such a constraint, only those who gain more than some limit can produce copies of themselves (reproduction is costly). The intention of this interpretation of environmental constraints with resulting social pressures to reproduce is that, throughout the iterations of the game, surviving strategies should aim to accumulate necessary resources and to occupy vital space in the limited population. Success in this context means that a strategy gains the resources necessary to make more copies of itself, i.e., to propagate its genes and memes; while losing means not being able to maintain the continuity of genes and memes of a given strategy. Thus, the simple PD begins to represent realistic evolutionary dynamics as constrained by available natural resources.

Some questions present themselves. If everyone can benefit with increased cooperation, how can this cooperation be guaranteed, and benefits realized? At the same time, how can players who want to benefit (and reproduce) without shouldering the burdens of cooperation (so-called free-riders) be avoided, with their reproduction being thereby limited, and their cost to the cooperating others in the society be minimized?

Minimizing opportunism while maximizing cooperation is a classic problem in Evolutionary Psychology [9]. This is a complex problem. Our approach to solving this problem involves using computers to simulate the evolution of diverse strategies in dynamic interaction with each other over multi-generation timespans. In the context of EGT, we design formal games combining cooperative and competitive situations with strategies that spontaneously may even mutate, until stable dynamics might emerge. One important aspect of such simulations that adds to their psychological realism is the capacity for agents not only to learn from each other, adapting to changing conditions by adopting successful strategies as exhibited by fellow constituents, but also to recognize strategies employed by one another through intention recognition, thereby helping to balance cooperative benefits with opportunism, as reviewed in the next section.

## 3. Intention Recognition

Intentions and their recognition play a central role in morality, as shown, for example, in studies on the moral doctrines of double and triple effects [24,28]. Several studies have focused on the evolution of cooperation in the context of EGT, viz. [3,29]. These, however, have neglected the role of intention recognition for cooperation in moral animals such as human beings, for whom learning how to recognize intentions and how to form mutual commitments accordingly can help solve cooperation problems [30,31,32]. The results of our research in this context, using the Iterated Prisoner’s Dilemma (IPD) [33], and employing tit-for-tat (TFT) and win-stay-lose-shift (WSLS) strategies, for example, show that, when groups include agents with some ability to recognize intentions, these will prevail against those that cannot. Moreover, intention-recognizing strategies result in significantly increased overall cooperation, even with extra cognitive costs associated with detecting a more or less hidden or an explicit intention. By focusing on these elementary though fundamental forms of cognition, plus social learning ability, the EGT approach affords a clearer view about the complex dynamics between the individual and the social collective, than what can be gained by approaches neglecting these fundamental cognitive abilities, and/or that proceed unaided by contemporary computational tools. Our work, hence, provides important insights into the reasoning of human moral agents. Moreover, by extension, it may also offer insights into how we may conceive of future moral machines as being able to recognize the intentions of others, perhaps thereby deserving consideration when we are confronted with decisions involving the design of such agents in the future.

## 4. Commitments

With intention recognition, moral agents can maintain higher levels of cooperative behaviour than without this capacity. In parallel, agreements cemented on prior communication should help to avoid misunderstandings. Usually, human beings establish commitments prior to beginning a cooperative project, for instance through a contract stipulating in advance the consequences for failing to deliver on a commitment. Commitments may be envisaged as intention manifestation, the obverse side of intention recognition. In corresponding research, we have been able to show that this capacity to form prior agreements may have evolved through natural selection, confirming the conclusions of [34]. In particular, we show that explicitly forming such agreements prior to cooperative action facilitates cooperation more effectively than just punishing afterwards, in spite of costs of forming such agreements.

Our studies use EGT to show that the cost of making commitments (e.g., paying an attorney to draw a contract) can be justified by overall higher payoffs for all, if the penalties paid to those who honour commitments by those who fail to do so are high enough, but without being too high. With such policies in place, agents representing strategies pursuing cooperative agreements become dominant, resulting in levels of cooperation unachievable in the society otherwise. Free-riders, those who intend to exploit efforts of cooperators for selfish and relatively short-sighted gains, are more easily avoided if intentions can be recognized or manifested. Interestingly, if penalties for breaking agreements reach a level approximate to the cost of forging a prior commitment plus the cooperation benefit, then no additional benefits result from increasing those penalties above this threshold. The implication here is that it is not necessary to enforce excessive penalties for violations of commitments, as such would induce undesired side effects, including cooperators becoming unwilling to bear the costs of forming such commitments. Rather, there is a “sweet spot” that balances the benefits of cooperation with the costs of free-riding opportunists, thereby helping solve the evolutionary dilemmas of cooperation.

As we have reported, intention recognition and the ability to form and to maintain prior commitments shed new light on the effects of these and other mechanisms on the evolution of cooperation. Moreover, greater levels of cooperation result from the formation of prior commitments [32]. Costs of contracts promoting cooperative behaviour are justified, in time and other resources, so long as they result in increased mutual social benefits beyond these costs. Our work thus shows that the ability of the individual agent to recognize intention plays an important role in promoting the emergence of cooperation at the level of the group. Indeed, by applying lessons from experience to current observations, the ability to recognize intentions facilitates cooperation, even without formal commitments like contracts. Recognition of intention alone is advantageous in avoiding costly commitments with free riders. However, to achieve higher levels of cooperation in a mixed and dynamic society where intentions cannot be evaluated with enough precision and confidence, formal agreements may still be necessary.

## 5. Punishment

The combination of selective commitment through intention recognition plus punishment of failure to honour prior commitment can prevent antisocial behaviour better than through intention recognition alone. In establishing this fact, our research group has made several comparisons between models, involving prior commitment with subsequent punishment of free-riders. Furthermore, we have compared these results with a strategy that does not first form prior agreements, but more simply just punishes offending parties after the fact.

Punishment does encourage cooperation in populations of merely self-interested individuals. However, necessary punishments of sufficient intensity can be so costly to punishers, and restrictions on those punished can be so excessive that significant levels of cooperation cannot be achieved, for such policies effectively remove opportunities for future cooperation after corrective measures are imposed. In particular, our studies show that arranging prior agreements establishing expected punishments for non-cooperation is able to reduce the costs of punishment enough for higher levels of cooperation to emerge. Interestingly, with such a policy in place, the cost of enforcing punishments declines at the same time.

Observing that prior commitments and punishments of failures to honour such commitments complement each other, we have researched how these strategies can synergize in order to deal with different types of dysfunctional behaviours. First, our research has been able to demonstrate that a probabilistic simple combination of both increases cooperation above what either alone can achieve when applied just by themselves. Effectively, the establishment of a prior commitment diminishes the cost–effect ratio relative to the benefit, should punishment need to be executed. This result turns out to be especially significant when the cost of creating such an arrangement is sufficiently low.

Our models have indeed shown that combining prior commitment with punishment leads to a significant increase in cooperation, overcoming the weaknesses of each strategy when exercised alone. However, what should one do when traitors can punish cooperators, as in forms of so-called anti-social punishment, such as those that may occur in the case of organized crime? [35] This is a long-standing problem and a major challenge in studies on cooperation evolution. Using the one-shot PD in which players engage in a single interaction, and in which some can propose cooperation agreements before an interaction as well as punish defectors afterwards, Han [35] showed that cooperation and social (as opposed to anti-social) punishment can co-evolve, even under the threat of anti-social punishment. With social punishment, only those who fail to honour a prior commitment to cooperate are obliged to pay compensation. As a result, anti-social punishers are significantly restricted by social commitment proponents. Still, because the cost to engage in social commitment agreements can be high, such regimes can be dominated by social punishers, for these do not shoulder the costs of forming commitments, all the while maintaining cooperation amongst one another.

Our results suggest that when punishment and commitment strategy options are both available in a population partly composed of anti-social players, a significantly greater cooperation is achieved than if only one of them is available, with an interesting caveat. The prior commitment mechanism alone strongly encourages cooperation but can be vulnerable to anti-social behaviour. The added payment of an extra cost of forming and enforcing commitments on top of mere punishment, which is vulnerable to anti-social punishers’ betrayal, is conducive to a significant increase in social cooperation and a decrease in anti-social behaviour. Thus, given the contribution to social welfare that this improvement represents, our research points to an interesting and important evolutionary dynamic, namely that forming prior commitments catalyses the emergence of the social punishers, and together the two mechanisms deliver greater benefit than either one by itself.

## 6. Public Goods

When considering cooperation problems, at root there is concern for public goods, especially about how to restrict free-riders from access to goods and how to monitor performance in honouring commitments without undue cognitive costs, whereby tracking free-riding behaviours, especially in large diverse populations, might add to uncertainty rather than help to manage the public good. Modern institutions, such as social health systems, work when prior agreements to contribute to them are formed and when commitments to doing so are honoured. Problems arise when free-riders (which do not contribute) take advantage of such agreements. Eventually, with too many free-riders, members of the group who initially commit to contribute will cease promotion of the public good in question, and the benefit that the public good would represent can be lost. In any such scenario, evident both in the natural world [34] and in laboratory experiments [36], prior cooperative commitments are essential to motivate cooperative behaviour in large diverse populations over the long term.

In the scope of a Public Goods Game (PGG) employing EGT to model large groups involving especially beneficial public goods, e.g., social health care, [37] showed that implementing measures to limit benefits to free-riders results in better social outcomes, even if these measures incur extra costs for cooperators. PGGs are standard frameworks used to study the emergence of cooperation within diverse groups of interaction [3]. PGGs involve players in groups of a fixed size. Each player can choose to cooperate, thereby contributing to the public good, or not to cooperate, thus not contributing to the public good. Choosing to not cooperate, a player thus becomes a free-rider who aims to gain selfishly from the contributions to the public good of those who chose to cooperate at the time the free-riders chose not to.

Indeed, in modelling such a PGG scenario, total constituent contributions to the public good are first multiplied by a constant factor representing the overall social benefit of cooperation. The resulting product is then distributed equally amongst all, regardless of whether any individual initially contributed or not. Injustice as unfairness arises due to the costs of forming commitments, for the individual cooperators. Cooperators gain less than non-cooperators who do not incur such costs, so free-riders are considered “immoral”. Moreover, should free-riders be permitted to avoid commitments while still gaining from the efforts of those who do, then cooperation is discouraged in subsequent iterations, since relative benefits for cooperators is diminished.

To further investigate commitment strategies, we expanded the PGG as described above in [38]. Before the actual start of a fold of plays, those PGG players wanting to commit to cooperation and contribute to the public good, demand that other co-players commit similarly and share the costs of setting up the agreement. A real-world example might involve paying costs associated with a formal contract specifying terms of mutual commitment. If co-players pledge to share associated costs, then public good proponents proceed on the assumption that everyone who so committed will contribute accordingly. If a player commits only after the initial agreement is established, and the public good goes forward, then that player has not contributed to the costs of initially forging the contract. The expectation, then, is that such a player must compensate those who did bear the burden of those costs and, as commitment proponents may encounter others who seek to avoid such costs while still benefitting from the resulting cooperative arrangement, strategies are required to address the negative impact of these individuals.

AVOIDANCE is the simplest such strategy and amounts to not creating the public good to start with. This strategy diminishes benefits that can be realized by those who seek to establish the public good, resulting in a moral dilemma. Alternatively, access to a created public good can be limited, so that only those who commit to paying associated costs enjoy (better) conditions of access to it. Or benefits for non-contributors might be reduced. This alternative is the RESTRICTION strategy. Our investigations, comparing these two strategies, deliver two main lessons:

(i) In a one-shot PGG, if costs associated with forming commitments are minimal relative to its benefits, both strategies encourage cooperation, thereby generalizing results of pairwise interactions to the larger group context;
(ii) As groups grow larger and/or as public good benefits increase, RESTRICTION encourages greater commitment to contributing to the public good, even when costs associated with restriction are quite high relative to the public good benefit.

In [39,40] we investigated commitment in PGGs differently, employing a different set of strategies. In summary, this work considers that constraints based on overall social benefit may not always be possible. Instead, many public goods can only be realized with a minimum level of participation. In such a context, agents demand prior commitments from other group members and agree to contribute themselves, based on how many of those others do choose to commit. With this information, agents can then calculate whether sharing in associated implementation costs is worthwhile, relative to overall social benefit. This research evinced that cooperative behaviour was encouraged when paying costs associated with forming prior commitments was voluntary but was contingent on minimal participation. Moreover, when costs are small enough, prior commitments are formed more frequently, resulting in greater cooperation within a population than when such costs are relatively high. Importantly, optimal levels of participation emerge depending on context. With increased costs associated with contributing to the public good, and with increased costs associated with the formation of prior commitment to contribute to said good, a higher degree of explicit prior commitment is necessary to ensure that benefits are realized.

Furthermore, in [39,40], we also considered that agents often delegate the formation of prior commitments as well as the monitoring of participation to an external other, e.g., an attorney, policeman, or institution, rather than attempting to undertake these efforts individually. In summary, in such a scenario, external delegated agents themselves also benefit with increasing cooperation, for example in the form of social services, including public transportation, institutional agreements between nation-states, or crowd-sourcing services. Delegated agents and institutions often require payment from committed group members for such services in the form of taxes and other fees. This research was able to show that such an approach to forming and enforcing commitments affords benefits beyond that of strategies depending on individual commitments and monitoring thereof. Instead of relying on constituents to take the initiative independently, issues involving unfairly distributed personal costs in forming and enforcing commitments, which prevent the personalized approach from realizing necessary levels of cooperation, can be eliminated. Again, this work confirmed that the level of participation is critical in deciding whether a prior agreement to cooperate towards a public good should be forged. Specifically, more rigour is required for an agreement to be formed through a centralized system, with its associated increased costs. However, once in place, such arrangements result in higher levels of cooperation, and increased benefits in terms of social welfare, due in part to a more wide-spread distribution of costs associated with forging and enforcing group-wide commitments.

## 7. Apology

Apologies often do not come easily, but our work finds that they do promote cooperation, especially in contexts that may be characterized by their uncertainty. In complex and changing situations, in attempting to coordinate action with diverse others with equally diverse aims, agents can make mistakes. Individuals who are able to apologize ensure cooperation, subject to the understanding that apology for present and future failure to uphold commitments to cooperate is costly, with its associated costs borne personally by the offending agent [41,42].

Apology is a ubiquitous mechanism reconciling conflicts between individuals during long-term repetitive interactions (e.g., in the context of marriage). Moreover, apology is possible without external actors, like those discussed in the previous section on public goods, namely parents, teachers, or courts, which would have a greater cost for all concerned. Apology is pervasive in real-world social human environments, like medical malpractice and seller–customer situations, where common experience associates apology with restoration of good will and therewith increased cooperation. It also facilitates positive emotions in online markets and human–computer interactions. Our research into the synergy between apology and commitment has shown that apologies are uncommon in interactions between participants without commitments to one another, especially if engaging in these cooperative apology interactions is costly. Our work shows instead that the formation of prior commitments increases the frequency of apologies. Moreover, our work has shown that apology can only rectify prior failures to honour commitments if sincere, with “sincerity” reflected as significant costs for the apologizing agent. Interestingly, because the lack of such costly apologies promotes free-riders with false commitments, our models correspond with real-world experience, in that more costly apologies are best exercised in more committed rather than less committed relationships.

In our above-mentioned work, we have used the Iterated Prisoner’s Dilemma (IPD) game to model populations which include agents embodying strategies that apologize between moves, subsequent to a mistake. An act of apology in this context consists of compensating the offended player to ensure that this player will cooperate, not defect, during the next move. With an adequate apology cost, a population of agents able to apologize can cooperate perfectly. Yet, strategies exploiting apologetic behaviour can emerge, including accepting compensatory apologies from others without apologising for one’s personal mistakes. Offering excuses, “false apologies”, without compensation for failures to maintain commitments, however, diminishes the overall benefits of apology for the group.

Our research using EGT has shown that, if apologies are executed in complement with the formation of prior commitments before the playing interactions start, the strategy of false-apology can be discouraged. This research generates the following main conclusions:

(i) Apology alone does not result in high levels of cooperation in a population;
(ii) Apology in complement to the formation of prior commitments results in much higher levels of cooperation than with either by itself;
(iii) Confirming other studies, e.g., [43], only sufficiently costly, i.e., “sincere”, apologies encourage cooperation, both in non-committed and in committed relationships;
(iv) Costlier apologies emerge in the context of committed relationships, with less-costly apologies suiting non-committed interactions, a result that we interpret as emerging because costlier apologies identify committed partners to the exclusion of free-riders and agents issuing false commitments, underscoring the principle that “commitments bring about sincerity.”

In sum, our studies confirm the importance of apology and commitment mechanisms in dynamic social settings, and help inform what type of apology may be appropriate in different real-world contexts, such as after mistakes in the context of a committed business relationship, for example, and if (and by how much) an apology should be augmented with commitment to future cooperation, like the compensation for irregularities suffered in the context of service provider-customer relationships, for example. Finally, once adequate apologies are offered, then there arises a complementary mechanism securing future cooperation when such apologies are accepted, namely through forgiveness, which is the subject of the next section.

## 8. Forgiveness and Revenge

So far, we have seen that making prior commitments proves to be a cooperative stable strategy—that is, one that if adopted in a population to a degree will continue to exist in it—in dilemmas of a single iteration involving all its members. Moreover, we have seen that cooperation also occurs in the context of long-term, repeated interactions amongst committed partners—that is, members of a group who will have to interact with each other repeatedly for long periods of time, even intergenerationally, rather than in the context of single, one-off, interactions. With this in mind, we have seen that apology emerges as a strategy to rectify mistakes in the execution of prior agreements to cooperate. In everyday human interactions, along with apology, we also witness capacities for forgiveness and for revenge.

In the context of EGT, we employed the IPD to determine under which conditions revenge and forgiveness emerge, alongside apology, to moderate cooperative agreements [42,44]. From this work, we learn, in summary, that sincere apology induces higher levels of cooperation and committed relationships even when mistakes are frequent, and that agents employ revenge, in the absence of apology, to defend themselves from and to discourage future defections. Hence, this work shows that revenge, apology, and forgiveness (accepting an apology, on evidence of the acceptance of future commitments to cooperate) play fundamental roles in inducing cooperation in repeated dilemmas.

In greater detail, some complexities are worth discussing, as they present themselves even in the context of relatively simple, structured interactions as modelled with the IPD. For one thing, commitments to cooperate may have to end before recurring interactions are realized. With this in mind, agents should embody strategies covering contexts in which an agreement is absent or present, as well as when such agreements should be proposed, accepted or rejected. For another thing, in contexts involving direct reciprocity [45], whenever mistakes are made, due to ‘confused minds’ or ‘trembling hands’, which add to the ‘noise’ of commitments in repeated interactions (that is, the imperfections of communication channels), agents must choose to take such mistakes as defections, thereby imposing a penalty that expects compensation, but forgiving mistakes after the issuance of a sincere (that is, costly) apology, and to proceed according to the prior agreement in the absence of an apology.

Our work proceeded with the understanding that behaviours including revenge and forgiveness emerge so that mutually beneficial cooperative relationships can continue in the face of mistakes. Consider revenge. Revenge, understood as an imposed cost either directly, with a fine or some other punishment, or, indirectly, by restricting access to some potential benefit, can discourage doing harm to others, including failures to honour commitments. However, in the context of noisy interpersonal relationships, typical of real-world interactions, one often cannot easily distinguish with sufficient certainty whether another’s erroneous behaviour is intentional or accidental. In such cases, revenge may be inappropriate, and forgiveness and apology may play fundamental roles.

As we have seen, forming and committing to agreements to cooperate is a basic mechanism moderated by different strategies in ongoing social interactions. For instance, our research has been able to show that the detrimental effect of high costs of agreement is moderated by apology and forgiveness, since an ongoing commitment can provide benefits over the course of multiple interactions, even in the face of mistakes due to different reasons, some of which may not be clear. With mistakes apparent, our work shows that apology, with sufficient costs incurred on the erroneous agent, is an essential ingredient for forgiveness. Furthermore, this research shows that, in the case of an accidental breach of prior commitments, forgiveness given adequate apology serves to restore relationships in order that cooperative interactions can take place in the future.

Given the above abilities, the agents who are most successful in a population are those that propose commitments, agree to pay costs of forming these commitments, and cooperate unless a mistake is made due to noise. Moreover, these agents enact revenge (retaining the transgressors’ share of the benefits from the agreed cooperation) and, most interestingly, will cheat during subsequent interactions. Revenge can deliver better outcomes than reciprocity (viz., the strategy tit-for-tat) in the IPD. Equally intriguing is the complementary result that forgiving agents do better only when the ratio between cost of commitment and benefit from cooperation is sufficiently large.

Now, since mistakes are inevitable in relationships in realistic (noisy) environments over the long-term, agents must determine whether or not to proceed according to a prior cooperative commitment when a co-player’s mistake is detected, and then to take compensation immediately or to forgive the other, that is, maintaining the agreement despite prior, current and inevitable future errors, which may be ascribed to noise. In order to study how agents moderate such interactions, in summary, in [42,44] we extended and detailed the model of commitment (introduced before) with apology and forgiveness. In this work, we defined apology as either an external parameter (say, defined by the Law), or as an individualized parameter (say, defined by peers). In either case, experiments confirmed that forgiveness restores cooperative relations if coming after an apology from the offending agent, though only if that apology is costly or “sincere” enough, but without being so costly as to impede the ability of the mistaken player to engage in future cooperation. Interestingly, this result is confirmed by experimental psychology and the Law [14,43,46,47,48].

The extension of our basic model of commitment with apology and forgiveness results in increased levels of cooperation above those achievable with revenge alone. However, we now know this result holds only if there is a minimum cost representing sincerity. Somewhat counter-intuitively, offered apologies that are less costly than this actually reduce the level of cooperation below that expected with simple revenge. Even more surprisingly, if there is minimal sincerity of an apology or it is not costly enough, then individuals will deceptively propose or accept commitments so as to take advantage of ongoing social agreements by failing to honour commitments and offer excuses (based on, e.g., confused minds or unsteady hands) in order to be forgiven, thereby gaining from cooperation without bearing its associated costs, and to end up dominating the population. Hence, without a minimal sincerity cost of apology, forgiving mistaken agents removes the benefits of cooperation that commitments deliver by themselves.

In the above-mentioned research, we were able to demonstrate that false commitments to cooperate and reluctance to engage in cooperative agreements may result from mistakes that break prior commitments to cooperate. This research led to the conclusion that only the imposition of a strict ethics involving costly apologies that induce forgiveness can prevent such outbreaks. In a complementary way, commitments in repeated interactions in which they are recognizable as loyal, differ from commitments with subsequent failure compensations. Loyalty involves choice of partner(s) in cooperative interactions. Loyal individuals select partners with whom they have shared prior interactions. The work reviewed above does not consider such a mechanism, but shows that commitment encourages lasting relationships, especially when reinforced with forgiveness in the face of sincere apology, whenever inevitable mistakes occur in noisy environments, wherein agents are often plagued with uncertainty about the intentions of others in diversified populations.

## 9. Guilt

Machine ethics involving the capacity for artificial intelligence to act morally is an open project for scientists and engineers. One important concern is how to represent emotions that are thought to modulate human moral behaviour, such as guilt, in computational models. Upon introspection, guilt is present as a feeling of being worthy of blame for a moral offence. Burdened with guilt, an agent may then act to restore a blameless internal state in which this painful emotion is no longer present.

Inspired by psychological and evolutionary studies, we have constructed theoretical models representing guilt in order to study its role in promoting pro-social behaviour. Again, in the context of EGT using the IPD [49], we modelled guilt in terms of two features. First, guilt involves a record of transgressions which we formalized as a counter tracking the number of offences. Second, guilt involves a threshold over which the guilty agent must alleviate the strained internal state, in the case of our models, through apology, and also by involving self-punishment, as required by the guilty feelings, both of which affect the payoff for the guilty agent. With this work, we were able to show that cooperation does not emerge when agents alleviate guilt without considering their co-players’ attitudes about the alleviation of guilt too. In that case, guilt-prone agents are easily dominated by agents who do not express guilt or who are without motivation to alleviate their own guilt. When, on the other hand, the tendency to alleviate guilt is mutual, and the guilt-burdened agent alleviates guilt in interactions with co-players who also act to alleviate guilt when similarly burdened, then cooperation thrives.

As we have seen, socially beneficial apology behaviour emerges within the context of long-term commitments, assuming that prior agreements are made before the IPD begins and that compensation is given if an agreement is broken and an (adequately sincere) apology from offending partners is forthcoming. In such scenarios, simple apology results in a payoff advantage to the offending co-player. However, a simple apology is limited. Modelling guilt explicitly has allowed us to explore more extensive aspects of commitment-breaking behaviours, including the cumulative effect of wrongdoings or the use of anticipation to decide what to do in the context of a specific guilt level. Guilt is unpleasant, and people may resist doing things when anticipating feeling guilty about them. People will obey rules to avoid feeling guilty afterwards about breaking them. Guilt in this way acts not only a posteriori, but functions a priori as well, preventing harm as agents wish to avoid guilt and with it the necessity to alleviate guilt as wrongs accumulate. Anticipation of guilt thus leads to a norm conformity, even when retaliation is not expected to arise (as when we might get away with free-riding). Anticipating our own guilt can defeat the temptation to engage in harmful behaviours and, if the emotional cost of guilt were removed, then norm conformity might drop off dramatically.

In social dilemmas modelled using the PD, defection becomes the dominant strategy. Defectors do better than cooperators regardless of whether their partners defect or cooperate. In such a situation, it is rational for both parties to defect, even though mutual defection is often worse than (i.e., provides less benefits than) mutual cooperation (depending on the structure of the game). [45] speculated that mutual evolution has promoted the emergence of guilt because it makes defection less attractive, with motivation from guilt becoming the dominant strategy due to corresponding social benefits. Individuals may gain materially by defecting, but guilt causes emotional suffering, and it is this suffering that encourages cooperation regardless of material gain [45].

However, common sense stresses that feeling guilt for harm done to others only makes sense if one perceives that those others do not intend harm as well. War is a case in point, i.e., guilt may be inappropriate if one kills if only to stop the other from killing first. Hence, recognising others’ intentions must be considered in any (realistic) model of guilt. Our very first guilt model confirms this point. Where recognizing the intention of another is not considered, then feeling guilty about defections without regard for what others feel about their defections is self-defeating, as we shall see.

Modelling guilt first requires that it be formalized [49]. Our approach formalizes guilt as an aspect of an agent’s genotypical strategies, and is quantified in terms of a threshold, G. On this model, G ∈ [0, +∞], and guilt at a given time is characterized by a transient level of guilt, g (g ≥ 0). As the experiment begins, g for every agent is 0. An agent’s transient guilt level, g, increases by 1 when that agent performs some action that is considered wrong, like making a mistake that leads to breaking a prior commitment to cooperate. After a number of mistakes resulting in g reaching that agent’s threshold of guilt, g ≥ G, the agent is able to choose to (or not to) act to reduce guilt g below that threshold. This model retains the mechanism of guilt alleviation described above, whereby guilt can be alleviated by apologising to offended partners, or by suffering guilt as self-punishment when apology to offended partners is not possible. On our approach, apology need not result in a benefit for the offended other. Rather, we consider apology as an honest expression of guilty feelings, signifying commitment to future cooperation in the face of current or prior mistakes. In our model, the alleviation of guilt is costly, with this cost quantified in terms of γ, with γ ≥ 0. A guilty agent suffers for guilty feelings in a form of self-punishment, this suffering being represented by quantity γ, with g being decreased (i.e., by 1). According to this definition, agents can be characterized in respect of different guilt thresholds. Some may be incapable of suffering guilty feelings, so their G = +∞. Others may be extremely prone to guilt, suffering guilty feelings with any mistake, and so for them G = 0.

Employing the IPD, our work has shown that agents capable of guilt are not only evolutionarily viable, but that they come to dominate in populations mixed with other agents that do not express guilt. Moreover, our experiments show that guilt-capable agents can induce committed, long-term social interactions, with these relationships resulting in a greater social benefit. These investigations focused first on two extremes, with G = 0 and G = +∞. Results were generalized afterwards to agents for which G > 0 and with G always less than the number of iterations of the IPD, because if G were to be larger than this, then G could in practice be given as G = +∞. These models employed a stochastic evolutionary model that incorporated a frequency-dependent mechanism for selection, as well as for mutation, so as to investigate under which conditions guilt-capable agents are evolutionarily beneficial, resulting in stable long-term cooperative relationships with an increased mutual benefit.

Importantly, this work showed that, to be evolutionarily viable, a guilt-prone agent-genotype must act in view of the capacity for its game partners to also express guilt. The lesson from these experiments is that self-punishment by suffering guilt, without considering whether partners are also similarly guilt-affected, does not result in guilt becoming a dominant feature of individuals in the population. On the contrary, when defecting partners do not express guilt when they themselves do, then an agent should either not experience guilt or its guilt should be automatically alleviated, at no cost. Otherwise, guilt-prone agents would be exploited by non-guilt prone free-riders with respect to guilt.

Depending on how an agent handles guilt, i.e., that agent’s strategy, the agent may self-punish, affecting its fitness, plus changing future behaviour. In our first model of guilt employing the IPD agents prone to guilt were not sensitive to expressions of guilt when its co-players defected. One lesson from that work was that agents prone to guilt but which are insensitive to guilt proneness of co-players are easily taken advantage of by free-riders who are not prone to guilt, and as a result do not afford increased cooperation in the group as a whole. In our second improved model, also in [49], guilt-prone agents were responsive to capacities for co-players to express guilt similarly. From it, we learned that such guilt-prone agents did improve on cooperation, overall. This agent strategy to experience and to express guilt, all the while being sensitive to the presence of this capacity in others, was more successful, being mimicked by other members of the population and becoming pervasive.

In the IPD, agents evaluate each other based on their behaviours, to defect or to cooperate. In realistic contexts, human beings also consider how others come to these decisions. People, first, tend to trust others who cooperate without ever thinking about defecting over those who do consider defection as an option, and then choose against trusting them later. According to Kant, “In law a man is guilty when he violates the rights of others. In ethics he is guilty if he only thinks of doing so.” [50]. Such sensitivity to the thought processes of others who may consider cheating or deception as an option involves a further capacity to recognize intentions. Consistent with Kant’s observations, our research confirms that intention recognition plays a crucial role in moderating social interactions, even when any given intention is not carried out [30,31,51].

From a multi-agent perspective, including mixed social-technological communities encompassing potentially autonomous artificial agents, and invoking the so-called “value alignment” problem (for a recent review cf. [52]), our models confirm that conflicts can be avoided when morally salient emotions, like guilt, help to guide participants toward acceptable behaviours. In this context, systems involving possible future artificial moral agents may be designed to include guilt, so as to align agent-level behaviour with human expectations, thereby resulting in overall social benefits through improved cooperation, as evinced by our prospective work on modelling guilt, summarized in this section.

## 10. Counterfactual Thinking

Counterfactual thinking (CT) involves a capacity to construct possible causal sequences. Moral judgement involves a capacity to choose between these alternatives, whereby the pursuit of one option implies not pursuing the others. This type of internal deliberation requires a cognitive capacity for counterfactual reasoning such as that evidenced in counterfactual arguments [19]. Counterfactuals serve the analysis of possible futures in this way. From an agent-level perspective, they are prospective of the form “What if the situation were different, and I would pursue this other action?” They also serve to analyse past situations and choices, as an agent is possibly informed by experience gained from the pursuit of a chosen action in the past. In such instances, an agent imagines the gains or losses that, knowing what it knows today, would have resulted had alternatives to actions pursued been carried out instead, e.g., “What if I would have done this instead of that?” [10,11,53].

CT is in this respect a prerequisite for AI agents employed in the analysis of, for example, legal cases or in the evaluation of ongoing governance of such AI agents and their development. Moreover, given counterfactual reasoning in human beings, namely in making judgments and in evaluating choices, the question arises of how this ability can improve cooperation and generate consensus in populations of otherwise solely self-interested individuals. The results of our research using EGT suggest that CT has limited cost and impact in cooperation problems wherein coordination of actions is not necessary, but that CT results in significant increases in cooperation in coordination problems in the context of large populations, such as those typical of human social situations [54].

Counterfactual statements are conjoined conditional statements in which both the antecedent and consequent clauses are false [55,56]. Counterfactuals, possibility, and the hypothetical are part of the genesis of what there is, and what there is is what it is because it was otherwise than it could have been. Moral responsibility is intuitively captured by a certain kind of counterfactual test, one that compares how the world is after our actions, with how the world would have been if, contrary to fact, we had not performed those actions. Similarly, a moral agent with leisure to do so can reason about alternative actions which might improve the world or produce a greater good than those routinely and uncritically performed or that may be under consideration. With this in mind, it is uncontroversial to follow [57] in suggesting that morality including statements of Moral Law depend on counterfactual reasoning ([57] p. 371).

The counterfactual theory of causal relations is dominant in both Law and recent Philosophy. CT is intimately related to causation as a natural relation at the heart of scientific explanation. Moral responsibility supervenes on natural properties like causation, via the intention to bring about a certain situation through action, in cognizance of possible alternatives. Accordingly, we hold each other to be more blameworthy when we cause some evil, rather than, if and when, we merely try to cause it. At the same time, it is common to experience negative emotions when we have caused some harm even though we were not culpable. In such cases, it is not regret but guilt that disturbs us, as we judge ourselves to be blameworthy even in the absence of the power or opportunity to have done otherwise ([57] pp. vi, vii, [41,58,59]).

According to judgement dissociation theory, “upward counterfactuals” involve CT employed to avoid negative outcomes. Upward counterfactuals negate direct causes and negate conditions potentiating negative outcomes or, alternatively, by adding conditions that disable negative outcomes. One implicit implication here is that an actor can prevent an outcome in more ways than the said agent can cause it. Hence, self-implicating upward counterfactuals are likely to draw attention to blame-implicating actions.

Complementary research carried out in the context of prisoner populations shows that programs that stimulate upward counterfactual thinking in prisoners, focusing on their sentences, crimes, arrests, and convictions, do result in increased self-attributions of blame, including the enhancement of guilty feelings, with positive implications for rehabilitation [59]. Commensurately, consider one of our own EGT studies of guilt. In [49], evidence is provided that, in a population wherein there exists from the start a modicum of guilt-feeling agents, better cooperation emerges as the capacity for guilt spreads. This result points to the distinct advantage of training prison detainees in CT, for the (re-)consideration of their actions and alternatives, with a view to honing their moral sensibilities, speeding their conditional paroles, and improving their future behaviour once released back into the general fold.

Intuitively, one factor in choosing to perform one action or another is the anticipated regret for undesired outcomes resulting from personal agency. Most generally, regret theories imply that the attractiveness of an option cannot be evaluated without reference to the context of other available options. On such accounts, regret is a response to the counterfactual outcome of a choice, and in this context the knowledge that a decision maker expects to have about that outcome should affect the anticipation of regret. In the setting of game theory, for instance, this knowledge is represented by the knowledge of the payoff matrix of a game. With this knowledge, a player can evaluate possible alternative payoffs [58].

CT is also employed in the moral evaluation of an agent’s intentions prior to action, given that a different outcome would have resulted had a different action sequence been composed and pursued. In such a situation, a natural concern is if the intended result could have been realised, perhaps without morally repugnant side-effects, if a different action sequence had been pursued. In [5,6,11,53], we have examined how CT can be employed to clarify moral responsibility for past actions using EGT. Our research in this area exhibits how CT can be utilized prospectively to produce greater good while avoiding harm, given experience of the joint outcomes of one’s and another’s actions in abstract social games [54]. In short, agent strategies employing CT promote the evolution of cooperation in populations of agents comprising diverse interaction strategies.

As we have seen so far in the preceding review of research, in the context of EGT, accumulated benefits over the course of an evolutionary game are associated with the relative fitness of agent strategies and individuals revise their strategies over the course of the game to mimic those strategies whose agents demonstrate greater fitness [3]. Accordingly, strategies that result in greater accumulated benefits proliferate. In more realistic settings involving agents such as human beings with robust capacities for CT, rather than be limited to simply copying successful strategies, such agents may employ native capacities for CT to formulate alternatives directly, without needing to wait for these alternatives to be demonstrated by other agents. Using CT, agents may envision how an outcome may have been improved if only they could have used more successful alternatives and would have chosen to pursue such alternatives, instead of having performed the actions they did. Endowed with robust capacities for CT, such agents can learn by themselves through reflection, and revise strategies without being constrained by mere imitation of available exemplars. This is where CT plays an important role in our ongoing work.

In [54], for example, we studied the effects on cooperation in a population of mixed strategies where some agents were able to creatively employ alternatives through such counterfactual reasoning. We compared the results of these trials with those involving a population constrained by social imitation alone. These were PPG experiments employing the famous “Stag-Hunt” coordination game [60] in which a minimal number of cooperative participants is necessary for the realization of social benefits. This game structure reflects natural and human social situations in which increasing levels of participation result in (non-linearly) increasing benefits. With this work, we sought to answer three focal questions:

1. Can counterfactual behavioural revision be formalized for large populations, adopting cooperation dynamics as a case study application?
2. If, instead of evolutionary dynamics with social learning, individuals now revise choices via counterfactual thinking, will cooperation emerge in collective coordination dilemmas?
3. Will there be a larger overall cooperation level impact when hosting a fraction of counterfactual thinkers in a social learners’ population? Will such a diversity in learning methods benefit cooperation?

Results delivered positive answers to all three questions. In the end, this work was able to show that players employing CT can have a profound impact on levels of cooperation. In summary, this research work was able to show that, even with a small proportion of a population able to employ CT in the formation of alternatives, without relying on social imitation to change strategies, and with the remaining population limited to social imitation, significant improvements in cooperation and associated benefits can be realized relative to a standard case in which the entire population is limited to social imitation.

## 11. Regulation of AI Safety Development and AI Race

In subsequent work [16,21], we have further utilized the methods of EGT to address the issues raised by the so-called AI development race and the potential risks posed by AI advance technologies, employing some of the mechanisms presented above, such as incentives and commitment [61,62]. AI is going through a period of great expectation, introducing a certain level of anxiety in research, business and also policy. This anxiety is further energized by an AI race narrative that makes people believe that they might be missing out. Whether real or not, a belief in this narrative may be detrimental as some stakeholders will feel obliged to cut corners on safety precautions or ignore societal consequences just to “win” [18]. We developed an EGT model that describes a broad class of technology races, where winners draw a significant benefit compared to others (such as AI advances, patent race, pharmaceutical technologies) [16]. We have also investigated how the reward of safety development and sanctioning of the unsafe one may beneficially influence outcomes. We uncovered conditions in which punishment is either capable of reducing the development speed of unsafe participants or has an untoward capacity to reduce innovation through over-regulation. We have shown that, in several scenarios, rewarding those that follow safety measures may increase the development speed while ensuring safe choices. Moreover, in the latter regimes, rewards do not suffer from the issue of over-regulation as is the case for punishment. These findings provide valuable insights into the nature and kinds of regulatory actions most suitable to improve safety compliance in the contexts of both smooth and sudden technological shifts.

However, there is yet another issue that needs to be solved for suitable regulation of AI development, because even if one can assess the timescale of the development, one still needs to estimate the measures of risk and gain associated with risk-taking behaviours. For that we need data, which are usually not yet available at an early stage of development. We have proposed an approach to solve this based on the idea of voluntary safety agreements. That is, over-regulation can be overcome if the race participants have the freedom to choose between pursuing their course of actions or arranging a mutual binding agreement to act safely. Punishment can then be carried out only against those that do not honour an adopted commitment [22].

## 12. Overall Conclusions

The preceding overview of studies, employing fundamental statistical methods to model population dynamics in the context of EGT, focused on insights gained employing public goods games and others, including the one-shot and iterated social dilemmas, relative to the emergence of cooperation and coordination, when enhanced by individual cognitive abilities, including capacities for intention recognition, for commitment, for apology and forgiveness, for guilt, and for counterfactual thinking, among others.

The methods used enable the formal identification of the entropic behaviours of populations of agents, by showing to which evolutionary stable states populations evolve, and under which initial conditions, ongoing rules of interaction and strategies.

In the end, this work justifies the fundamental status afforded moral capacities in theories of social agency, demonstrating that these capacities are necessary in order to identify false commitments and free-riders, to recognize others’ intentions, to assume and detect guilt, or to creatively compose socially optimal coordination strategies by oneself, through self-reflection, by means of counterfactual thinking. We have also started to employ some of these moral capacities and mechanisms to provide insights into the emerging issues of AI regulation and governance, including how reward, punishment, and commitments, setting up a baseline framework to apply in the future other mechanisms such as guilt, intention recognition and counterfactual thinking. Formal specifics involved with the analytical modelling, the simulation details and their quantitative results can be found in select references to our published work offered in this presentation.

Finally, social and juristic challenges of artificial intelligence [63] might benefit from utilizing some of our modelling forays.

## Figures and Tables

**Table 1:** Prisoners’ Dilemma.

	Prisoner B: Silence	Prisoner B: Confesses
Prisoner A: Silence	Prison for six years, for each of them	A: Prison for ten years B: Prison for two years
Prisoner A: Confesses	A: Prison for two years B: Prison for ten years	Prison for eight years, for each of them

## Data Availability

No data involved. This is an overview article, with no new results.
